# Risk of Intrapartum Cervical Lacerations in Vaginal Singleton Deliveries in Women With Cerclage

**DOI:** 10.14740/jocmr2227w

**Published:** 2015-07-24

**Authors:** Shunji Suzuki

**Affiliations:** Department of Obstetrics and Gynecology, Japanese Red Cross Katsushika Maternity Hospital, 5-11-12, Tateishi, Katsushika-ku, Tokyo 124-0012, Japan. Email: czg83542@mopera.ne.jp

**Keywords:** Cerclage, Cervical lacerations, Vaginal delivery

## Abstract

**Background:**

We examined the obstetric outcomes of singleton vaginal deliveries in women with cerclage at our institute to confirm the risk of intrapartum cervical lacerations in vaginal deliveries of women with cerclage.

**Methods:**

Data on all Japanese singleton vaginal deliveries at ≥ 34 weeks’ gestation managed at the Japanese Red Cross Katsushika Maternity Hospital between 2008 and 2014 were collected.

**Results:**

During the study period, cervical cerclage was performed in 95 of 9,490 (1.0%) women with singleton pregnancy at 12 - 22 weeks of singleton pregnancy who delivered at ≥ 34 weeks’ gestation. The incidence of intrapartum cervical lacerations and postpartum hemorrhage ≥ 1,000 mL in the women with cerclage were higher significantly than that in the women without cerclage (cervical lacerations: crude odds ratio (OR): 26.9, 95% confidence interval (CI): 14 - 51, P < 0.01; postpartum hemorrhage: crude OR: 2.86, 95% CI: 1.6 - 4.9, P < 0.01). Using a multivariate analysis, cerclage was independently associated with the increased incidence of intrapartum cervical lacerations (P < 0.01).

**Conclusions:**

Cervical cerclage is an independent risk factor of intrapartum cervical lacerations in vaginal deliveries.

## Introduction

To date, the benefits of cervical cerclage for cervical insufficiency to reduce the incidence of preterm birth have been well investigated [1, 2]. However, cerclage has been reported to be associated with some obstetric complications such as intrapartum cervical lacerations due to cervical scarring [3-6]. In this study, we examined the obstetric outcomes of singleton vaginal deliveries in women with cerclage at our institute to confirm the risk of intrapartum cervical lacerations in vaginal deliveries of women with cerclage.

## Methods

The protocol for this study was approved by the Ethics Committee of the Japanese Red Cross Katsushika Maternity Hospital. Our hospital is one of the major perinatal centers in Tokyo, Japan (about 2,000 deliveries per year). Informed consent concerning analysis from a retrospective database was obtained from all subjects.

Data on all Japanese singleton vaginal deliveries at ≥ 34 weeks’ gestation managed at the Japanese Red Cross Katsushika Maternity Hospital between 2008 and 2014 were collected. In our institute, the McDonald or Shirodkar cerclage is performed with a sterile plastic suture (Matsud Suture^®^, Japan) at 12 - 22 weeks’ gestation for women with history- or ultrasound-indicated cerclage by informed consent. We usually remove the suture at 34 - 35 weeks’ gestation.

Demographic information and the characteristics of the labor were extracted from patient charts to examine the obstetric outcomes associated with cerclage. In this study, we examined the maternal age, parity, body mass, smoking, alcohol consumption, gestational age at delivery, duration of labor, oxytocin use, severe perineal lacerations: perineal laceration either third- or fourth-degree laceration, cervical lacerations requiring suture, postpartum hemorrhage, neonatal birth weight and umbilical artery pH. Precipitous labor was defined as expulsion of the fetus within less than 3 h of commencement of regular contractions.

Data are presented as number (%). For statistical analysis, the χ^2^ test for categorical variables was used. Odds ratios (ORs) and 95% confidence intervals (CIs) were also calculated. Differences with P < 0.05 were considered significant. A multivariate logistic regression model, with backward elimination, was constructed in order to find independent factors associated with cerclage.

## Results

During the study period, cervical cerclage was performed in 95 of 9,490 (1.0%) women with singleton pregnancy at 12 - 22 weeks of singleton pregnancy who delivered at ≥ 34 weeks’ gestation. The McDonald suture was performed for 88 women, while the Shirodkar suture was performed for seven women with history- or ultrasound-indicated cerclage. All sutures were removed at 34 - 35 weeks’ gestation.

Table 1 shows the clinical characteristics and obstetric outcomes in the patients with and without cerclage who delivered at ≥ 34 weeks of singleton pregnancy. The rate of nulliparous women, smokers and abnormal vaginal deliveries in the women with cerclage were lower significantly than those in the women without cerclage (nulliparity: crude OR: 0.21, 95% CI: 0.12 - 0.35, P < 0.01; smoking: crude OR: 0, P = 0.04; abnormal deliveries: crude OR: 0.33, 95% CI: 0.12 - 0.69, P = 0.02). The incidence of precipitous labor in the women with cerclage was higher significantly than that in the women without cerclage (crude OR: 2.00, 95% CI: 1.3 - 3.1, P < 0.01). The incidence of intrapartum cervical lacerations and postpartum hemorrhage ≥ 1,000 mL in the women with cerclage were higher significantly than that in the women without cerclage (cervical lacerations: crude OR: 26.9, 95% CI: 14 - 51, P < 0.01; postpartum hemorrhage: crude OR: 2.86, 95% CI: 1.6 - 4.9, P < 0.01). There were no other significant differences in these valuables between the women with and without cerclage.

**Table 1:** Clinical Characteristics and Obstetric Outcomes in Women With and Without Cerclage Who Delivered at ≥ 34 Weeks of Singleton Pregnancy

	No cerclage	Cerclage	P-value
Total	9,395	95	
Maternal age ≥ 40 years	692 (7.4)	9 (9.5)	< 0.01
Nulliparity	4,804 (51.1)	17 (17.9)	0.21
Previous cesarean deliveries	43 (0.5)	0 (0)	0.51
Smoking	387 (4.1)	0 (0)	0.04
Alcohol consumption	120 (1.3)	0 (0)	0.27
Body mass index ≥ 25 at pre-pregnancy	1,259 (13.4)	7 (7.4)	0.09
Preterm birth at 34 - 36 weeks	413 (4.4)	4 (4.2)	0.93
Vacuum/forceps/breech delivery	1,114 (11.9)	4 (4.2)	0.02
Duration of delivery < 3 h	1,973 (21.0)	33 (34.7)	< 0.01
Duration of delivery ≥ 24 h	11.2 (1.2)	0 (0)	0.90
Oxytocin use	2,053 (21.9)	13 (13.7)	0.05
Severe perineal lacerations	264 (2.8)	3 (3.2)	0.83
Cervical lacerations	55 (0.6)	13 (13.7)	< 0.01
Postpartum hemorrhage ≥ 1,000 mL	553 (5.9)	14 (14.7)	< 0.01
Neonatal birth weight < 2,500 g	820 (8.7)	11 (11.6)	0.32
Neonatal birth weight ≥ 3,500 g	860 (8.7)	7 (7.4)	0.55
Umbilical artery pH < 7.1	153 (1.6)	1 (1.1)	0.66

Data are presented as number (percentage).

Using a multivariate analysis, cerclage was independently associated with the increased incidence of intrapartum cervical lacerations (adjusted OR: 21.1, 95% CI: 10 - 43, P < 0.01).

## Discussion

Clinically significant intrapartum cervical laceration, defined as those associated with bleeding or requiring cervical suturing, is an uncommon complication of vaginal delivery [7]. To date, the high incidence of cervical lacerations has been associated with operative delivery (vacuum, forceps and breech deliveries), precipitous labor, nulliparity, induction of labor, and birth weight > 4,000 g [3-6]. In this study, the incidence of precipitous labor in the women with cerclage was higher significantly than that in the women without cerclage; however, the rate of other risk factors for cervical lacerations in the women with cerclage were lower significantly than that in the women without cerclage. Nevertheless, the current observations indicate the high risk of cervical lacerations in vaginal deliveries of women with cerclage.

In this study, the incidence of cervical lacerations in women with cerclage was 14%, compared with only 1% in the control women. The current results were similar to those in some previous studies [3-6]. The precise reason for the association remains unclear. It has been supposed to that the foreign body reaction around the cervical stitch and the resulting scar tissue make the cervix more susceptible to injury during delivery [3].

The current results may indicate the independent risk of cervical lacerations associated with cerclage; however, the current study may be limited because the relation between cervical insufficiency and intrapartum cervical lacerations has not been well documented. The incompetent cervix may be intrinsically more prone to injury. Recently, Seravalli et al [7] reported a retrospective cohort study in singleton pregnancies at high risk for preterm birth. In their study, the all cases were high-risk women who underwent either a history-indicated or ultrasound-indicated cerclage, and cervical lacerations occurred with similar frequencies in the women with and without cerclage (2.2% vs. 1.3%, P = 0.78). However, in their study the both incidences of cervical lacerations in the group with cerclage and preterm birth in the group without cerclage seemed to be low compared with some previous studies. Therefore, a further study concerning the relation between cervical insufficiency and lacerations may be needed.

### Conclusions

Cervical cerclage is an independent risk factor of intrapartum cervical lacerations in vaginal deliveries. The serious management is needed in the delivery of women with cerclage.
